# In vivo study of the behavior of glass ionomer restorations in patients with special needs

**DOI:** 10.4317/medoral.26537

**Published:** 2024-06-22

**Authors:** María Grau-Benítez, Francisco Javier Silvestre, Agustín Pascual, Alberto Albero, Javier Silvestre-Rangil

**Affiliations:** 1 Associate Professor, Department of Dental Materials, European University of Valencia, Valencia, Spain; 2 Professor. Department of Stomatology, University of Valencia, Valencia, Spain; 3 Assistant Professor, Department of Dental Materials, University of Valencia, Valencia, Spain; 4 Associate Professor, Department of Dental Materials, European University of Valencia, Valencia, Spain; 5 Assistant Professor, University of Valencia, Valencia, Spain

## Abstract

**Background:**

Glass ionomers may be a good alternative to composite resin restorations in special needs patients with challenging behaviours. The present study was carried out to evaluate the restorative efficacy of glass ionomer in the occlusal cavities of permanent molars among patients with special needs after one year of follow-up.

**Material and Methods:**

A randomized split-mouth study was made of a cohort of patients with special needs. First and second permanent molars with occlusal caries were treated with glass ionomer, silver amalgam and composite resin. Assessments were made at 3, 6 and 12 months, using a scale based on the original code of Ryge and the USPHS criteria.

**Results:**

A total of 34 patients and 102 restorations comprised the study sample. The survival rate of both the glass ionomer and silver amalgam was 100%, versus 97.1% in the case of composite resin. The glass ionomer afforded good marginal adaptation and sTable color, with no fractures or secondary caries.

**Conclusions:**

The glass ionomer remained successfully for one year in the occlusal cavities of the permanent molars, with the same survival rate as silver amalgam, and better survival than composite resin, in the patients with special needs.

** Key words:**Glass ionomer, dental caries, disability, special needs patients.

## Introduction

Good restorative treatment for caries seeks to prevent progression of the lesion;preserve the non-demineralized and remineralizable tissue;secure adequate sealing with the preservation of pulp vitality, thereby restoring tooth function and aesthetics;and avoid the accumulation of plaque upon the surface (1).

Silver amalgam has traditionally been used as a restorative material in patients with special needs thanks to its durability, good mechanical properties, lesser dependency upon the technique employed, and the successful results obtained in patients at a high risk of developing caries (2). Since silver amalgam does not adhere to the tooth, relatively invasive mechanical retaining cavity designs are required when using this material. Other inconveniences of silver amalgam are its deficient aesthetic effect and the presence of mercury in its composition (3). For this reason, many countries have decided to reduce the use of silver amalgam, and an agreement was reached at the Minimata Convention on Mercury in 2013 to gradually eliminate the use of this durable restorative material (4).

The growing demand for more aesthetic and mercury-free restorations has led to an increased use of composite resins. Indeed, in recent years, composite resins appear to have become the material of choice in direct restorations. The data found in the literature increasingly confirm the durability of composite resins as restorative material, though their application has some inconveniences. In effect, total isolation of the tooth is required, and a stepwise material placement technique is advised (2). In the case of patients with special needs, these requirements may represent an important obstacle, due to the operating time required and the fact that difficulties in correctly performing the technique (poor opening of the mouth, macroglossia, challenging behavior, salivation or repeated head movements on the part of the patient) can result in vacant spaces within the restoration or may even leave some non-polymerized zones at the base or between the different applied layers. This in turn can result in diminished resistance, defective sealing of the restoration, postoperative sensitivity, or early failure of the restoration (5).

The main advantages of glass ionomer in these circumstances include its biocompatibility, intrinsic adhesion of the material to both the enamel and dentin, and the continuous release of fluoride - which contributes to remineralization and even inhibits or reduces plaque accumulation (3). Likewise, glass ionomer is easy to handle in difficult circumstances and tolerates a moist environment, since a degree of humidity is required during the material setting process (3). Furthermore, glass ionomer is characterized by a low thermal expansion coefficient similar to that of the natural tooth, biocompatibility and low cytotoxicity. However, the mechanical properties of GICs, such as compressive strength, tensile strength, and hardness, could limit their clinical applications. In addition, in the early stages of setting, the material is sensitive to moisture and desiccation. It is the low resistance to abrasion or wear that led to the incorporation of other substances to the classical composition of GICs (resins and metals) in order to increase their values and expand their applications. Reinforced VICs offer greater resistance to wear, compression and traction when compared to non-reinforced ones (6). In this regard, the new restorative glass ionomers may be a good alternative to composite resin restorations (7) and could prove to be valuable in special circumstances (8) characterized by deficient patient cooperation and/or tolerance of dental treatment - offering the dentist a new simple and effective tool for restoration treatments in patients of this kind (3).

The present study was carried out to evaluate the restorative efficacy of glass ionomer in the occlusal cavities of permanent molars among patients with special needs after one year of follow-up. Maintenance of the anatomical shape was assessed, together with color stability, staining of the restoration, staining of the margin, marginal adaptation, the presence of secondary caries, fractures in the restoration, and retention of the restoration. The null hypothesis was: “After 12 months of service, glass ionomer occlusal restorations in permanent molars of special needs patients don’t have inferior success compared to silver amalgam and composite resin in relation to their durability. The anatomical shape is adequate, the marginal adaptation is sufficient for the follow-up period with no differences in color stability, staining, probability of secondary carious lesions, risk of fracture and loss of restoration retention.”

Materials and Methods

A randomized, prospective, longitudinal split-mouth study was made of a cohort of patients with special needs. The restorative filler materials were randomly assigned to each tooth needing restorative treatment.

A random number generator from 1 to 3 was used. One of these numbers corresponded to each material, 1 being glass ionomer, 2 being composite, and 3 being silver amalgam. Following the order by quadrants (1, 2, 3 and 4), the material was assigned to the restoration of the first quadrant with a carious lesion, and so on with the rest.

The study was approved by the Research Ethics Committee of the University of Valencia (H1395331357926), and abided with the principles of the Declaration of Helsinki. The confidentiality of the patient data was maintained. All participants or their responsible persons signed an informed consent.

- Study population and selection criteria

A prior study of the necessary sample size was carried out. The results indicated that a minimum of 32 patients with 96 restorations were sufficient to achieve 90% power in order to detect an effect size f=0.15 (medium-small) as significant for the contrast of intra-subject effects (differences across over time or between types of material). A drop-out rate of 25% was anticipated, so an attempt was made to recruit at least 42 patients.

The study subjects were recruited on occasion of their first visit to the clinic for patients with special needs (Red Cross), and had been diagnosed with intellectual disabilities at their reference hospital center. Intellectual disability (intellectual developmental disorder) is defined as a disorder that begins during the developmental period and includes limitations of intellectual functioning as well as adaptive behavior in the conceptual, social, and practical domains.

The inclusion criteria were the presence of three occlusal carious lesions in permanent molars and a positive or definitely positive attitude according to the Frankl behavioral scale (9).

Patients presenting an American Society of Anesthesiologists (ASA) score of IV or V (according to the Medical Risk Related History (EMRRH) questionnaire) (10) were excluded, as were those showing little or no cooperation during clinical management, such as, for example, not remaining seated in the dental chair, aggressiveness, insufficient mouth opening for taking pictures or the impossibility of performing intraoral X-rays. On the other hand, patients with signs of bruxism, and smokers were also excluded.

The study in turn included first and second permanent molars with occlusal caries corresponding to International Caries Detection and Assessment System (ICDAS) code 3, 4 or 5 with the antagonist tooth. We excluded primary/deciduous molars, permanent third molars, teeth subjected to endodontic treatment, occlusal lesions not caused by caries, replacements of damaged or lost restorations, and molars with restorations of other surfaces.

- Restorative materials employed

**Figure 1:**
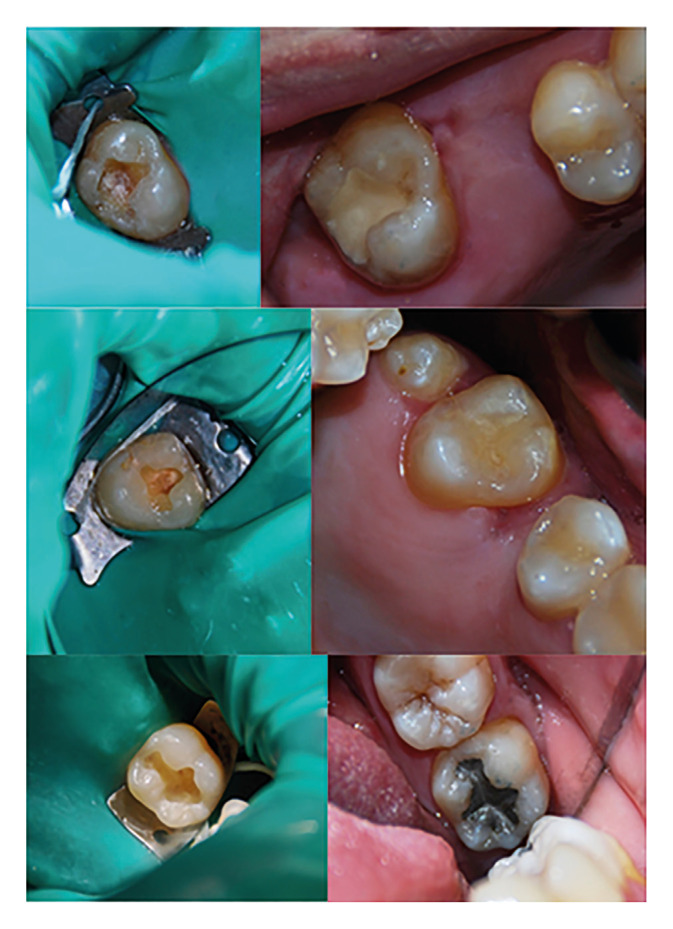
Examples of glass ionomer, composite resin and amalgam restorations and cavities.

All the restorations were made under conditions of total isolation by the same clinician. For cavity preparation, high and low speed rotary instruments with diamond drills were used and in their conFiguration they sought to be as minimally invasive as possible, only removing the part of the tooth damaged by caries in the case of composite resin and glass ionomer. In the design of the silver amalgam cavity, the general and traditional principles of cavity design were followed. In all cases, a medium-thickness latex rubber dam and Ivory® clamps were used. Prior the application of each material, all cavities were subjected to washing with 5.25% sodium hypochlorite (Fig. 1).

The composition of the materials used in the study is described in Table 1. We used regular setting, non-gamma 2 spherical particle silver amalgam (Tytin®, Kerr, MI, USA) supplied in pre-dosed capsules, with mixing performed according to the instructions of the manufacturer during 8 seconds in a Ventura Mix 2 amalgam vibrator (Madespa S.A., Toledo, Spain). For composite resin filling, we chose the TetricEvoCeram®BulkFill nanohybrid composite (Ivoclar Vivadent, Schaan, Liechtenstein) together with TetricEvoFlow®BulkFill fluid composite (Ivoclar Vivadent, Schaan, Liechtenstein), using the 37% phosphoric acid etching technique and Prime & Bond NT® adhesive (Dentsply Sirona, Konstanz, Germany).

The protocol was: acid etching for 15 seconds, washing with water and drying the tooth, then 2 layers of adhesive blown between them and photopolymerized both at the same time for 10 seconds. The composite resin was light-cured for 20 seconds.

Photopolymerization was carried out with a Bluephase photopolymerizing lamp (Ivoclar Vivadent, Schaan, Liechtenstein). For polishing we used a turbine-driven fine grain diamond bur with a counterangle Arkansas polishing bur.

As glass ionomer, we chose the EQUIA® self-setting, self-adhesive restorative system (GC Europe, Leuven, Belgium) supplied in pre-dosed capsules and activated following the instructions of the manufacturer, with an amalgam vibrator mixing time of 10 seconds. A capsule applicator was used for dispensing the material. No tissue conditioner was used prior to application of glass ionomer cement.

Monolithic block cavity filling was performed for subsequent condensation and modeling. Two minutes and 30 seconds after starting the mixture (following the instructions of the manufacturer), final finishing and polishing was carried out using the same burs as in the case of the composite resin, with occlusion adjustment for posterior application of the EQUIA coat® protective varnish (GC Europe, Leuven, Belgium) and photopolymerization during 20 seconds.

No commercial or publicity interests influenced our choice of the working materials, i.e., there were no conflicts of interest.

- Follow-up visits

All the clinical assessment of the restorations was performed by the same clinician. The follow-up visits took place 3 months (T1), 6 months (T2) and 12 months (T3) after placement of each restorative material and included visual and tactile inspection using a flat Hu-Friedy mirror and WHO periodontal probe. Photographic documentation of the restorations was compiled, and silicone impressions were obtained (putty consistency, regular and light) with a standard cuvette to obtain a model for subsequent indirect review.

The silicon impression positive was made with NOVOX® polyol resin (Dynamic Abutment Solutions, Lleida, Spain). The replicates were examined using a loupe magnifying system (ExamVision Galileo HD 3.8 x).

For ethical reasons, control radiographs were only obtained on occasion of the 12-month follow-up visit.

As clinical assessment criteria, we used the original code of Ryge, adapted to the more detailed descriptions afforded by Hickel *et al*. (11) and accepted by the FDI. In this way we adopted a scale more closely adjusted to the characteristics of the present study (Table 2), classifying the condition of the different restorative materials after 3, 6 and 12 months of follow-up, according to different clinical parameters.

- Statistical analysis

An inferential analysis was made based on the estimation of a Brunner-Langer nonparametric longitudinal data model for each response variable or clinical characteristic. The model included two intra-subject factors: the type of restorative material and the timepoint over follow-up. Analysis of variance (ANOVA) was used to assess main effects and interactions. The significance level in the different analyses was 5% (α=0.05). The statistical methodology used, with a confidence level of 95% and considering an effect size to be detected f=0.15 (medium - small), afforded a statistical power of 91% for the comparison of intra-subject effects. A prior study of the required sample size showed a minimum of 32 patients with 96 restorations to be sufficient to reach a statistical power of 90% in detecting an effect size f=0.15 as being significant for the comparison of intra-subject effects. A dropout rate of 25% was contemplated;the inclusion of at least 42 patients was therefore considered.

## Results

A total of 34 patients (13 females and 21 males) completed the follow-up period.

The scales used in this study have been designed to reflect the filling performance of three different restorative materials and to assess the aesthetic qualities of two of them.

The 12-month follow-up period was completed by 34 patients with special needs, with a total of 102 restorations. The patient recovery rate was 79.06%. At 12 months, the EQUIA® glass ionomer survival rate was 100%, versus 97.1% for the Tetric EvoCeram® BulkFill composite resin and 100% for Tytin® silver amalgam.

The details referred to the behavior of the different restorative materials at 3 months (T1), 6 months (T2) and 12 months (T3) are shown in Table 3.

- Analysis of the evolution of the clinical characteristics

Anatomical shape:

Both the composite resin and silver amalgam received an alpha score in 100% of the restorations at all follow-up timepoints. In the case of the glass ionomer, all the restorations received an alpha score at three and 6 months, while at 12 months of follow-up 91.2% maintained and alpha score and 8.8% received a bravo code. The glass ionomer restorations showed only a weak tendency (*p*=0.074) to present less than excellent scores.

Color stability:

The evaluation of color stability was made for the glass ionomer and composite resin, but not for silver amalgam. Both the glass ionomer and composite resin received an initial alpha score in 100% of the cases at all three evaluation timepoints (T1, T2 and T3).

Following evaluation of the photographs, the composite resin maintained an alpha score for all the restorations at T1, T2 and T3. In contrast, the glass ionomer received an alpha score in all cases at T1 and T2, though at 12 months, 97.1% of the restorations maintained an alpha score and 2.9% received a bravo code. There was no significant difference in the evolution of color between the two materials (*p*=0.317).

Staining of the restoration:

At three months of follow-up, the EQUIA® glass ionomer presented an alpha score in all restorations. At 6 and 12 months, an alpha score persisted in 97.1% of the cases, while 2.9% received a bravo code.

At the first and second evaluation timepoints, Tetric EvoCeram® BulkFill composite resin received an alpha score in 97.1% of the restorations and 2.9% received a bravo code. At 12 months of follow-up, 91.2%, 5.9% and 2.9% of the restorations received an alpha, bravo and charlie score, respectively.

Lastly, in the case of Tytin® silver amalgam, all of the restorations received an alpha score at three and 6 months, while at 12 months an alpha score persisted in 97.1% of the cases, and 2.9% received a bravo code.

There were significant changes in staining over follow-up (*p*=0.046), though the changes were similar for all three restorative materials (*p*=0.544).

Marginal staining:

The EQUIA® glass ionomer showed no marginal staining at three or 6 months, though at the end of the 12-month follow-up period an alpha score was recorded in 91.2% of the restorations, with a bravo code in 8.8%.

In the case of Tetric EvoCeram® BulkFill, clinical examination at three months revealed no marginal staining in any of the restorations;however, on evaluating the photographic records, an alpha score was recorded in 97.1% of the cases and a bravo code in 2.9% at this first evaluation timepoint. At both 6 and 12 months, 91.2%, 5.9% and 2.9% of the restorations received an alpha, bravo and charlie score, respectively. The latter corresponded to moderate staining at the margin that could not be eliminated through polishing and required repair.

In relation to silver amalgam, 100% of the restorations initially received an alpha score at all three timepoints. However, on reviewing the photographs, slight marginal staining was noted at 6 months in 2.9% of the cases and at 12 months in 5.9% of the cases, with the recording of a bravo code in both periods.

Thus, there were significant changes in marginal staining over follow-up (*p*=0.013), though the changes were similar for all three restorative materials (*p*=0.449).

Marginal adaptation:

In the case of the glass ionomer, all of the restorations showed excellent marginal adaptation at the first evaluation timepoint, and only 2.9% received a bravo code at 6 and 12 months.

The composite resin likewise received an alpha score at three months, though at 6 months 2.9% of the restorations received a charlie code and required repair. Lastly, at 12 months, 97.1% maintained an alpha score while 2.9% received a delta code - the latter implying replacement of the restorative material and repeat filling.

At three months, all the silver amalgam restorations showed excellent marginal adaptation. At 6 and 12 months, 97.1% maintained an alpha score while 2.9% received a bravo code.

Although the evaluation of marginal adaptation showed changes in level in some cases (*p*=0.074), it was homogeneous and common to all three types of restorative material (*p*=0.998).

Secondary caries:

In the present study, all the restorations made with glass ionomer and all the silver amalgam fillings presented an alpha score at all three evaluation timepoints, i.e., no recurrent caries were observed. However, in the case of the composite resin, 33 restorations were found to be in excellent condition at 6 months, with no secondary caries, though one of the restorations showed a small cavitation at the margin that could be repaired. In turn, at 12 months, 33 restorations continued to present no recurrent lesions, while one restoration showed secondary caries with exposed dentin in which repair was not possible, and replacement was thus required (delta code).

No changes were observed in relation to the presence of secondary caries - the findings being extrapolaTable to all three restorative materials (*p*=0.317).

Restoration fractures and retention:

None of the three materials suffered severe fractures implying loss of the restoration. In all cases, the final retention rate at 12 months was 100%. The only relevant finding was the identification of fine cracks in the glass ionomer of one restoration at 12 months that nevertheless did not affect marginal integrity (bravo code).

No changes were observed in relation to the presence of fractures or retention problems - the findings being similar for all three materials (*p*=0.317).

## Discussion

Few randomized, prospective, longitudinal split-mouth studies on the efficacy and survival of restorative materials in patients with special needs can be found in the literature. Restoration success has been defined as “the demonstrated ability of a restoration (including a prosthesis) to perform as expected. Restoration failure may be defined as any condition that leads to replacement. Conditions that constitute restoration failure include secondary caries, irreversible pulpitis, excessive wear, excessive erosion, unaccepTable esthetics, fracture, and bulk fracture”.

The present study confirms the restorative efficacy of glass ionomer during a period of at least one year in application to occlusal caries in patients with special needs.

The dental treatment of patients of this kind is complicated. As a result, the use of glass ionomer could be a valuable alternative in such situations where restorative treatment may prove difficult. In effect, glass ionomers are rapid and easy to handle;tolerate placement in a slightly moist environment (3);adhere to both enamel and dentin, saving time in preparing the cavity;favor continuous fluoride release that contributes to remineralization;and even inhibit or reduce plaque accumulation. In addition, glass ionomers are particularly useful for the atraumatic restorative technique, defining satisfactory clinical performance, and may be an alternative restorative option for occlusal cavities (12).

The continuous development of these materials has resulted in considerable improvement of their physical properties and in the placement technique (5), defining them as the option of choice in cases characterized by a high risk of caries (13). Glass ionomer may be a valuable alternative in special circumstances (8), as its use is less invasive than the traditional protocol and thus causes less patient discomfort. Furthermore, glass ionomer simplifies the restoration process and allows the dentin-pulp tissue complex to react against the development of caries (14). Thus, the use of this material not only improves dental treatment but also reduces patient morbidity by limiting the need for general anesthesia and sedation - thereby improving access to dental care in this patient population (15).

Different studies have described secondary caries as the most common reason for restorative treatment failure with aesthetic materials, though some authors consider that fewer recurrent carious lesions are observed at the margins of a glass ionomer restoration (16). We were able to confirm this in patients presenting important technical difficulties, since we recorded no secondary caries in the course of follow-up with either glass ionomer or silver amalgam - though such lesions were indeed observed in the case of composite resin.

According to the literature, microleakage is related to dimensional changes, and in this regard contraction phenomena with the use of glass ionomers must be taken into account. The oldest studies on the marginal adaptation of glass ionomers evidenced poorer outcomes than in the latest publications. This may be explained by the different materials used or by the evolution of the different products - since the manufacturers constantly introduce changes intended to improve their properties (17).

In the present study, glass ionomer maintained good marginal adaptation in patients in which the placement of this material proves more difficult. Its performance was similar to that of the other restorative materials, in coincidence with other authors who reported good outcomes with no need for intervention or marginal adaptation problems (18).

Glass ionomer appeared to be the material with the best behavior in terms of marginal staining, since no such phenomena were observed at either T1 or T2. Friedl *et al*. (18) found that only one out of 151 glass ionomer restorations presented distinctive marginal staining after two years in the mouth. Reviews after 10 years of follow-up have evidenced excellent conditions in 84% of the sample (19). Such manifest success may be related to the fluid coating used, which creates a regular surface and affords protection of the margins. This photopolymerizable coating aims to form a resin layer that seals and protects both the restoration zones and the adhesive interface between the restoration and the dental structure.

However, according to Pacifi *et al*. (20), the glass ionomer surface coated with the photopolymerizable nanofilling resin exhibits surface roughness similar to that seen in non-coated restorations. In addition, they point to the need for two additional steps (application on the surface of the restorative material and photopolymerization) as a potential inconvenience when it comes to treating scantly cooperative patients.

On the other hand, in our study, composite resin yielded poorer results in terms of staining of the restoration. The reasons for such poorer performance are not clear. Considering the technical sensitivity of this restorative material and the fact that patients with special needs might not be fully cooperative, the explanation may be defective polishing together with other factors related to diet or medication.

In our study there were no significant differences in the evolution of the color of the glass ionomer when compared with composite resin. This observation is similar to that reported by Gurgan *et al*. (19), who found color concordance with EQUIA® in class I cases to be excellent after one year in the mouth.

Gao *et al*. (21) reported scant color consistency with glass ionomers, which darkened over time. The authors related this to wearing of the material, increasing surface roughness and staining of the material and of the margin. In contrast, Diem *et al*. (22) noted no significant differences between the materials, and even reported improvement of the color of the glass ionomer restorations over time (22,23).

With regard to maintenance of the anatomical shape, a recent study found silver amalgam to remain superior to all the other examined materials, thanks to its metallic nature (24). Other publications have concluded that composite resins could replace silver amalgam, since some of them exhibit wear rates similar to those of silver amalgam (25).

In relation to glass ionomer, some studies describe much greater wear in comparison with silver amalgam or composite resin (26), and report that despite the improvements afforded, resin-reinforced ionomers still suffer some loss of anatomical shape and surface wear, particularly over the middle and long term (17).

In our study there were no significant differences in the anatomical shape variations between composite resin and glass ionomer. We only recorded a slight tendency to deviate from the original condition - though without clinical consequences. Other authors likewise have reported no significant differences (27), and indicate that utilization of the G-Coat Plus photopolymerizing varnish avoided volume loss of the glass ionomer restorations (18).

The varnish coating appears to afford protection against early wear of the restoration (22) until the material becomes fully mature and resistant. Nevertheless, disappearance of the coating has been described over time as a result of wear caused by chewing (23).

Studies on the reasons for failure of the three restorative materials report that 12% of the glass ionomer fillings are replaced due to loss of anatomical shape, versus 9% of the composite resin restorations and none of the silver amalgam fillings (27).

Lastly, in relation to the presence of fractures and/or loss of retention of the restoration, none of the studied materials suffered severe fractures implying loss of the restoration. Some authors indicate that part of the adult population with special needs may suffer weakened chewing force associated to systemic disease conditions that could reduce the restoration failure rate (28). In contrast to this, bruxism in some patients with special needs probably plays an important role in the appearance of cracks or fractures in the restorations (29). This aspect would constitute a limitation of our study, for although the patient sample was carefully selected, differences in chewing force or undiagnosed parafunctional habits could have influenced the final outcome. Another limitation is the one-year duration of the study. In this regard, longer follow-up periods would be needed to evaluate the behavior of these materials over the middle to long term. All patients were treated at the Red Cross dental clinic, since it was a community clinic, the material available at that time was used.

Based on the results of our study, glass ionomer appears to be a suiTable material for the restoration of carious lesions in patients with special needs. Furthermore, considering the technical difficulties posed by these patients and the impossibility of securing an adequate field for the placement of composite resin restorations, glass ionomer may be seen as a treatment alternative in those patients who otherwise would require measures such as sedation or general anesthesia.

Although glass ionomer has been widely studied and is popular in conservative dentistry, further prospective longitudinal studies are required in patients with special needs. This restorative material is very useful in scenarios characterized by poor patient cooperation, and offers the dentist a new and effective tool for the dental restoration of patients of this kind.

It can be concluded that glass ionomer remains successfully in the mouth for one year in occlusal cavities of permanent molars, with the same survival rate as silver amalgam and a greater survival rate than composite resin, in patients with special needs.

A weak tendency to deviate from clinical excellence is observed in relation to maintenance of the anatomical shape and color stability. As regards staining and marginal adaptation, the observed changes are similar with all three of the studied restorative materials.

It is important to underscore that we observed no secondary caries, fractures or loss of retention of the glass ionomer restorations during the studied period, with performance similar to that of silver amalgam and superior to that of composite resin.

## Figures and Tables

**Table 1:** Composition of the materials used in the study.

Tipe of material	Material	Manufacturer	Composition
silver amalgam	Tytin^®^	Kerr	Mercury, silver, tin, copper.
composite resin	Tetric EvoCeram^® ^BulkFill	Ivoclar Vivadent	Bis-GMA, Bis-EMA, UDMA, barium aluminum silicate glass, ytterbium fluoride, mixed spherical oxide, prepolymers, additives, catalysts, stabilizers, pigments, camphorquinone, Ivocerin®.
	Tetric EvoFlow^® ^BulkFill	Ivoclar Vivadent	
Glass ionomer	EQUIA^®^	GC Europe	Polyacrylic acid, strontium fluoroaluminosilicate glass, distilled water.
Low viscosity resin coating	EQUIA coat^®^	GC Europe	Methyl methacrylate, colloidal silica, camphorquinone.

**Table 2:** Dental restoration evaluation criteria.

Clinical characteristics	Alpha	Bravo	Charlie	Delta
Anatomical shape	Ideal shape	The shape differs slightly, but is not aesthetically unpleasant. Lack of material without dentin exposure.	The shape is affected, with an aesthetically unacceptable outcome.Lack of material with dentin exposure.Correction / intervention is necessary.	The shape is completely unacceptable or is lost. Replacement is required.
Color stability	Good color stability. No difference in tone or translucency.	Slight deviation. Does not affect aesthetics and is acceptable.	Localized but clinically unsatisfactory deviation that can be corrected through repair.	Unacceptable. Replacement required.
Staining of the restoration	No surface staining.	Slight surface staining (dry conditions) without being aesthetically unacceptable, and extending over all the teeth. Polishing required.	Extensive staining of the restoration surface. Aesthetically unacceptable. Correction required.	Severe and / or sub-surface staining. Replacement required.
Marginal staining	No staining between the restoration and the tooth.	Slight marginal staining (dry conditions). No adverse aesthetic impact.	Moderate staining, but located at the margin and not eliminated by polishing. Correction required.	Severe generalized and deep staining. Replacement required.
Marginal adaptation	Harmonious contour, without gaps or decoloration.	Small marginal fracture that can be corrected by polishing.	Gap < 0.5 mm that can be repaired.	Gap > 0.5 mm with fractured enamel / dentin. Replacement required.
Secondary caries	No secondary caries.	Very small and localized demineralization. No treatment required.	Cavitation / localized caries that can be repaired.	Deep secondary caries or exposed dentin. Repair is not possible and the restoration must be replaced.
Fracture and retention	Restoration retained without fractures / cracks	One or two fine cracks that do not affect marginal integrity.	Fractures that impact upon marginal quality.Affecting less than half of the restoration.	Partial or complete loss of the restoration.

**Table 3:** Restoration material behavior at 3, 6 and 12 months post-treatment.

Clinical characteristic		Glass ionomer			Composite resin			Silver amalgam		
		3 months	6 months	12 months	3 months	6 months	12 months	3 months	6 months	12 months
		n_r _=	n_r _=	n_r _=	n_r _=	n_r _=	n_r _=	n_r _=	n_r _=	n_r _=
Anatomical shape	Alpha	34	34	31	34	34	34	34	34	34
	Bravo	-	-	3	-	-	-	-	-	-
	Charlie	-	-	-	-	-	-	-	-	-
	Delta	-	-	-	-	-	-	-	-	-
Color stability	Alpha	34	34	33	34	34	34	-	-	-
	Bravo	-	-	1	-	-	-	-	-	-
	Charlie	-	-	-	-	-	-	-	-	-
	Delta	-	-	-	-	-	-	-	-	-
Staining of the restoration	Alpha	34	33	33	33	33	31	34	34	33
	Bravo	-	1	1	1	1	2	-	-	1
	Charlie	-	-	-	-	-	1	-	-	-
	Delta	-	-	-	-	-	-	-	-	-
Marginal staining	Alpha	34	34	31	33	31	31	34	33	31
	Bravo	-	-	3	1	2	2	-	1	2
	Charlie	-	-	-	-	1	1	-	-	-
	Delta	-	-	-	-	-	-	-	-	-
Marginal adaptation	Alpha	34	33	33	34	33	33	34	33	33
	Bravo	-	1	1	-	-	-	-	1	1
	Charlie	-	-	-	-	1	-	-	-	-
	Delta	-	-	-	-	-	1	-	-	-
Secondary caries	Alpha	34	34	34	34	33	33	34	34	34
	Bravo	-	-	-	-	-	-	-	-	-
	Charlie	-	-	-	-	1	-	-	-	-
	Delta	-	-	-	-	-	1	-	-	-
Fracture and retention	Alpha	34	34	33	34	34	34	34	34	34
	Bravo	-	-	1	-	-	-	-	-	-
	Charlie	-	-	-	-	-	-	-	-	-
	Delta	-	-	-	-	-	-	-	-	-

n_r:_Number of restorations.
